# Peripheral position of *CCND1* and *HER-2/neu* oncogenes within chromosome territories in esophageal and gastric cancers non-related to amplification and overexpression

**DOI:** 10.1590/S1415-47572009005000034

**Published:** 2009-06-01

**Authors:** Lucimari Bizari, Eloiza Helena Tajara, Ana Elizabete Silva

**Affiliations:** 1 Departamento de Biologia, Universidade Estadual Paulista Júlio de Mesquita Filho, São José do Rio Preto, SPBrazil; 2 Departamento de Biologia Molecular, Faculdade de Medicina, São José do Rio Preto, SPBrazil

**Keywords:** * CCND1*, *HER-2/neu*, chromosome territories, esophageal carcinoma, gastric cancer

## Abstract

Interphase chromosomes have been shown to occupy discrete regions of the nucleus denominated chromosome territories (CTs), their active genes being preferentially positioned on the surfaces of these CTs, where they are accessible to transcriptional machinery. By means of FISH (Fluorescence *in situ* Hybridization), we analyzed the *CCND1* and *HER-2/neu* gene positions within the CTs and their relationship with gene amplification and protein over-expression in esophageal and gastric cancers. The *CCND1* and *HER-2/Neu* genes were more often positioned at the periphery (mean frequency of 60%-83%) of the CTs in tumor tissues of the esophagus and stomach. Moreover, this positioning revealed no association with either gene amplification or the protein over-expression status of these genes, although, in esophageal carcinoma, Kappa statistics showed a moderate agreement between amplification of the *CCND1* gene (Kappa = 0.400) and its location within the CT, as well as with over-expression of the corresponding protein (Kappa = 0.444). Thus, our results suggest that gene positioning in interphase chromosomes does not follow a definitive pattern neither does it depend only on gene transcriptional activity. Apparently, this positioning could be both gene- and tissue-specific, and depends on other factors acting together, such as dense-gene, chromosome size, chromatin structure, and the level and stability of its expression.

## Introduction

Studies focusing on the molecular components of DNA replication, transcription, RNA processing, DNA repair and the nuclear matrix (Berezney *et al.*, 1995; Lamond and Earnshaw, 1998) have demonstrated that the inter-phase nucleus is structurally and functionally compartmentalized into regions that contain factors involved in RNA synthesis, processing and transport (Park and De Boni, 1998). However, except for the nucleolus, little is known of how various nuclear structures arise and influence gene expression (Nunez *et al.*, 2008).

Chromosomes have been shown to maintain their identity during the entire cell cycle with moderate movement during the interphase (Zink *et al.*, 1998), thus occupying discrete, non-overlapping regions of the nuclear volume called chromosome territories (CTs) or chromosome domains (CDs) (Cremer *et al.*, 1993; Visser and Aten, 1999).

Particular emphasis has been placed on the question of whether the transcriptional activity of genes can affect their territorial positioning and, indeed, whether active genes can leave the territory altogether (Bartová *et al.*, 2000; Williams, 2003; Harnicarová *et al.*, 2006). Kurz *et al.* (1996) were the first to demonstrate that in muscle cells, fibroblasts and HeLa cells, the *DMD, HBB* and *MYH7* genes (but not non-coding regions) are preferentially located in the periphery of their respective CTs, regardless of their activity. Extending this analysis, other studies have reported the preferential positioning of different genes in the periphery of the CTs in certain cell types, with or without association with transcriptional activity (Dietzel *et al.*, 1999; Volpi *et al.*, 2000; Galiová *et al.*, 2004; Scheuermann *et al.*, 2004; Harnicarová *et al.*, 2006). On studying colon cancer HT-29 cell lines, Harnicorová *et al.* (2006), revealed that both normal and amplified *c-myc* genes, when inactive, are positioned preferentially in the internal region of chromosome 8. However, *c-myc* transcripts associated with the site of synthesis are located more peripherally.

In contrast, Mahy *et al.* (2002) found that the *WAGR* locus at 11p13 containing the genes *WT1, RCN, PAX6* and *PAXNEB,* as well as intergenic non-transcribed DNA, is located within the painted chromosome 11 territory and, more importantly, that transcriptional activation of these genes did not result in their relocation to the surface of the territory. These authors suggested that active genes might be found not only on the surface, but also within the territory and on the surfaces of the condensed chromatin sub-domains that line invaginating interchromatin channels.

It is important to note that, to date, only a limited number of genes/loci have been examined in terms of intra-territorial or intra-nuclear distribution and their potential relationship to gene expression. Therefore, it is unclear as to whether all genes behave similarly (Kosak and Groudine, 2002). Moreover, there is evidence that genes can be found anywhere within a chromosome territory, regardless of their transcriptional activity (Parada *et al.*, 2002), and that the positions of gene loci with respect to territory are not absolute but can represent a statistically significant percentage. Thus, the core question is whether particular types or classes of genes demonstrate a conserved territorial position and whether cancer cells show modified nuclear architecture, thereby suggesting a functional relationship between nuclear organization and gene expression (Zaidi *et al.*, 2007).

Given the controversy arising from positioning active transcriptional sequences in the periphery of chromosome territories, the aims of the present study were to evaluate spatial intranuclear distribution of *CCND1* and *HER-2/neu* oncogenes within their respective chromosome 11 and 17 territories in esophageal carcinoma, gastric adenocarcinoma and normal mucosae, and to correlate the results so obtained with the gene amplification and protein over-expression status of both the *CCND1* and *Her-2/neu* genes. Esophageal and gastric cancers were chosen as models due to indications of increased gene amplification and over-expression of *CCND1* and *HER-2/neu* sequences.

**Figure 1:**
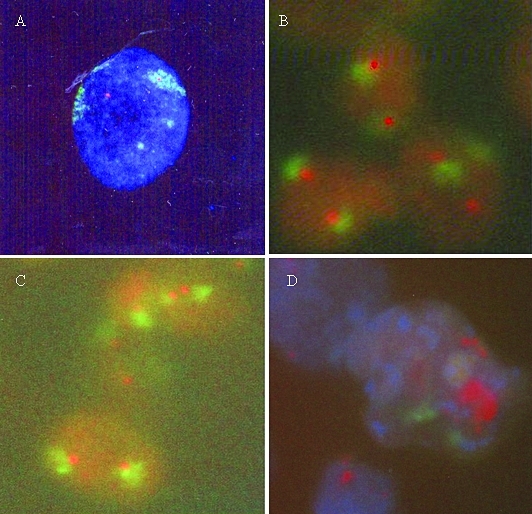
Fluorescence *insitu* hybridization (FISH): (A) *CCND1* gene (red signal) in the internal region of chromosome 11 territory (green) in normal esophageal mucus; (B) *CCND1* gene (red signal) located at the periphery or external to chromosome 11 territory (green) in gastric adenocarcinoma; (C) *HER-2/neu* gene (red signal) located at the periphery or external to chromosome 17 territory (green) in esophageal carcinoma: (D) *CCND1* gene amplification (red cluster) located in the external region of chromosome 11 territory (green) in esophageal carcinoma.

## Material and Methods

### Samples

Twenty specimens of fresh tumor tissue were obtained from 10 patients with esophageal squamous cell carcinoma (all males) and 10 patients with gastric adenocarcinoma (8 males and 2 females), prior to any form of chemotherapy and/or radiotherapy treatment. All the patients were diagnosed and operated on for esophageal and gastric cancer by surgeons of the Department of Surgery of the *Hospital de Base*, in São José do Rio Preto, SP, Brazil. The mean age of the patients with esophageal carcinoma was 58.2 years (range 46-65 years), and that of the patients with gastric adenocarcinoma was 56 years (range 38-82 years). The esophageal tumors were graded according to the World Health Organization, whereby 9 tumors were classified as moderately differentiated and 1 as adenocarcinoma. The gastric adenocarcinomas were classified according to characteristic histological features (Laurén, 1965) into diffuse (7 specimens) or intestinal (3 specimens) types. Biopsies (5 of normal esophageal mucus and 5 of normal gastric mucus) obtained from these same patients but distant from areas of the recent surgery, were used as controls.

The project was approved by the National Research Ethics Committee (CONEP), and written informed consent was obtained from all patients.

### Cell culture

Esophageal and gastric cancer and normal mucous tissues were cultured, with modifications, according to the Volpi *et al.* (2000) protocol. Briefly, the cells were cultured in an α-MEM medium, supplemented with 10% fetal-calf serum, 1 mM sodium pyruvate, 0.1 mM non-essential amino acids and penicillin/streptomycin, in an incubator at 37 °C with a humidified atmosphere containing 5% CO2. After about 15-20 days, the cells were synchronized in the G0/G1 phase by serum deprivation for 48 h. The cells were then detached with trypsin, washed by centrifugation in an α-MEM medium, and incubated in a hypotonic solution (0.075 M KCl) for 20 min at 37 °C. These were then fixed by standard cytogenetic procedure in three changes of fresh 3:1 methanol:acetic acid and placed onto clean glass slides. Although methanol:acetic acid generates a looser chromatin packing, thus allowing for only bi-dimensional analysis, it has been used successfully to study large-scale chromatin organization *in situ*, with results comparable to paraformaldehyde fixation and three-dimensional preservation of the nuclear structure (Yokota *et al.*, 1997; Bartová *et al.*, 2000; Volpi *et al.*, 2000; Galiová *et al.*, 2004). In fact, it has been shown to provide an alternative method, with quicker analysis than confocal microscopy and 3-D reconstruction.

### Fluorescence in situ hybridization (FISH)

#### Chromosome territories

Dual-color FISH assays were performed on cell suspensions obtained from the cell culture, using the LSI Cyclin D1 sequence labeled in SpectrumOrange, Whole Chromosome Painting (WCP) 11 labeled in SpectrumGreen (Vysis), the HER-2/neu kit (Q-bioGene), including the *HER-2/neu* sequence probe labeled with Rhodamine, and WCP 17 labeled with Fluorescein, all according to manufacturer's instructions. Briefly, the slides were treated with 70% acetic acid at room temperature for 5 min. A solution containing either Cyclin D1 and WCP11 or HER-2/neu and WCP17 probes was then applied to the specimens and co-denatured at 80 °C for 5-8 min. Subsequently, the slides were hybridized overnight (16 to 18 h) at 37 °C in a moist chamber. After post-hybridization washing, these were then counterstained with DAPI (4', 6-diamino-2-phenylindole), where upon chromosomal territories (#11 and #17) were visualized in green and genes (*CCND1* and *HER-2/neu*) in red.

The slides were examined -on an Olympus BX60 microscope. The positions of FISH signals from individual regions (*CCND1* and *HER-2/neu* genes) of chromosomes 11 and 17 were analyzed in relation to respective chromosome territories, and defined by means of chromosomes 11 and 17-specific paint into approximately 200 territories, according to previously reported protocol (Volpi *et al.*, 2000). Each territory within polysomic nuclei was evaluated separately. In this analysis, the gene signal was named “internal” if it appeared inside the painted domain but not touching its border, “peripheral” if touching the border and “external” if outside the painted domain without touching the border. In our experiments, both “peripheral” and “external” hybridization patterns were grouped together under the one definition “peripheral”, due to the low frequency of external signals.

### Gene amplification

FISH assays were performed on cell suspensions obtained from non-cultured fresh tumors and normal tissues, by using the Cyclin D1 probe kit (Vysis) including the LSI Cyclin D1 sequence labeled in SpectrumOrange with the chromosome 11 centromere sequence labeled in SpectrumGreen and the HER-2/neu kit (Q-bioGene) including the probe HER-2/neu sequence labeled with Rhodamine with the chromosome 17 centromere labeled in Fluorescein, according to manufacturer's instructions and previously described protocol (Bizari *et al.*, 2006).

These slides were also evaluated through an Olympus BX60 microscope. The number of signals from the chromosome 11 centromere and that from the Cyclin D1 gene were scored together, the same occurring with the corresponding number from the chromosome 17 centromere with that from the *HER-2/neu* gene, in around 250 nuclei and in accordance to criteria described by Eastmond *et al.* (1995). The gene-to-centromere (G/C) ratio was calculated in each case. In other words, the total number of gene signals observed was divided by the total number of centromere signals. For each FISH probe tested, the status of the chromosome used as control was classified as disomy or polysomy when, respectively and on an average, either 2 or 3 or more copies were scored per nucleus. Gene gains were considered either by the amplification or by high-balanced polisomy that is recognized as over-representation (Sunpaweravong *et al.*, 2005). Gene amplification assessed by multiple copies of the gene in any chromosomal status was classified into a high level group when either the gene:centromere ratio was > 2.0 or when it consisted of clustered copies (groups of many copies of the gene), while high-balanced polisomy was considered positive only in cases of high chromosomal number frequencies (> 15%).

### Immunohistochemistry (IHC)

Tumor material consisted of paraffin-embedded specimens fixed in 10% formalin. Sections were sliced 5 μm thick and dried for 1 h at 65 °C. After having been de-paraffinized and rehydrated, antigen retrieval was carried out in a Tris-EDTA solution pH 9.0 for Cyclin D1 (clone DCS-6, Novocastra, 1/100) and 10 mM citrate pH 6.0 for the Her-2/neu (clone 5A2 Novocastra, 1/100) antibody. All further steps were undertaken using an automated system (Cadenza) and according to protocol described by Bizari *et al.* (2006). Tonsil and breast carcinomas were used as positive controls for Cyclin D1 and the Her-2/neu antibodies, respectively.

All specimens were revised by one single pathologist, whereby only the IHC status was evaluated. Approximately 500 nuclei were counted for Cyclin D1 immunostaining. The positivity index was defined at values greater than 30%, and with 20% of the nuclei showing strong Cyclin D1 immunostaining in both esophageal carcinomas and gastric adenocarcinomas, according to the mean frequencies observed in the respective normal mucus. The intensity and pattern of membrane staining of the HER-2/neu antibody considered for scoring were in accordance with criteria approved by the FDA (Food and Drug Administration): 0 - no membrane staining observed in less than 10% of tumor cells; 1+ - partial membrane staining in more than 10% of tumor cells, but no circumferential membrane staining; 2+ - weak to moderate circumferential staining observed in more than 10% of tumor cells; and 3+ - pronounced circumferential membrane staining observed in more than 10% of tumor cells. Scores 2+ and 3+ were considered positive. Cytoplasmatic immunostaining was not taken into consideration for either protein. Areas that were poorly preserved, crushed, cauterized, folded or retracted were specifically avoided.

### Statistics

Two-tailed ANOVA statistical test, using a Statidisk computer software program, was performed to compare mean frequencies of gene position between normal and tumoral tissues with arcsin (p)^1/2^ transformation. The level of significance was set at p < 0.05.

Two-tailed Kappa statistics was carried out to evaluate the agreement between gene location in the CTs and gene amplification or over-expression levels for the two molecular targets. Agreement Analysis by Kappa was done, using an on line tool (Lee, 1995) Kappa values and interpretation were undertaken according to the Landis and Koch (1977) classification.

## Results

Summaries of the results of the location of both genes in the chromosome territories, their amplification and protein over-expression are presented in Tables 1  and 2. In all the experiments, peripheral and external patterns were grouped together within the category ‘peripheral', in view of the low frequencies of the external position (mean range 1.4% to 12.2%) observed in most cases. Regarding the *CCND1* gene, about 50% of the signals were found to be present either inside (Figure 1A) or at the periphery of painted chromosome 11 territory in normal mucus of both the esophagus and the stomach, whereas in esophageal and gastric carcinomas, higher frequency was observed in the periphery of the CT (60.55% and 83.73%, respectively) (Figure 1B). In fact, the ANOVA test only showed significant differences in mean frequencies of *CCND1* gene positions between the groups ‘normal mucus' and ‘gastric cancer (p < 0.0001). Interestingly, a different pattern was found for the *HER-2/neu* gene, since about 60% of the signals were located in a peripheral position in chromosome 17 territory (Figure 1C) in the esophageal carcinoma, as well as in normal gastric mucus and gastric adenocarcinoma samples, whereas in the normal esophageal mucus, this was found preferentially in an internal position (66.54%). Statistically significant differences were found between the group of gastric cancer (p = 0.0017) and the respective group of normal mucus. No large external chromatin loop was observed in any of these cases, although, a larger signal was evident in a cluster outside the chromosome territory in some of those amplified (Figure 1D).

The association of *CCND1* and *HER-2/neu* gene locations within the respective chromosome territories, together with gene amplification and over-expression of these two molecular targets, is shown in Tables 3  and 4 for esophageal carcinoma and gastric adenocarcinoma, respectively. According to Kappa statistics, no exact association between these parameters was found in either esophageal or gastric cancers, but only a moderate agreement between amplification of the *CCND1* gene and internal or peripheral positioning of this gene within its respective CT in esophageal carcinoma (Kappa = 0.400; p = 0.088). Regarding over-expression, moderate and slight poor agreement of Cyclin D1 and Her-2/neu protein expression with gene positioning was also observed in esophageal carcinoma (Kappa = 0.444; p = 0.091 and Kappa = 0.194; p = 0.301, respectively).

## Discussion

Nuclei are structurally and functionally compartmentalized, with their active genes positioned on the surface of chromosome territories, where they are accessible to transcriptional machinery (Cremer *et al.*, 1993; Visser and Aten, 1999; Volpi *et al.*, 2000). Disorganization of such a structure might play a major role in certain human diseases, such as cancer (Guasconi *et al.*, 2005). Therefore, both chromatin modification and the positioning of genetic loci in the nucleus play critical roles in control-gene expression (Cremer and Cremer, 2006). This model appears to be true for at least some gene/loci. Nevertheless, studies on gene positioning in different cell types are still scarce and the results conflicting. Understandably, it is therefore unclear whether all genes behave in the same manner or not.

The aim of this study was to analyze inter-phase chromosomes in nuclei of primary tumor cell cultures in an attempt to verify whether there is a preferential position for the different genes within the chromosome territory itself, and to understand the relationship between gene positioning and its respective activity. As a model system, we examined *CCND1* and *HER-2/neu* oncogenes in normal mucus and tumoral tissue of both the esophagus and stomach by using a FISH assay, since these genes have been shown to become amplified and/or over-expressed in these two types of tumor (Takehana *et al.*, 2002; Shiomi *et al.*, 2003; Dahlberg *et al.*, 2004; Manoel-Caetano *et al.*, 2004). The FISH technique has been found to be a powerful tool for investigating the nuclear organization of specific chromatin regions (Hepperger *et al.*, 2007).

In this study, *CCND1* and *HER-2/neu* genes that did not present a common pattern were often detected near the periphery (peripheral and external positions) of their chromosome territories. This was mainly so in tumors in comparison to normal mucus.

Cytological preparations were prepared by using cell fixation according to standard cytogenetic procedures, thereby generating a looser chromatin packaging and permitting only 2D analysis. This procedure has been successfully used in previous studies to examine large-scale chromatin organization *in situ,* with results comparable to those achieved with paraformaldehyde fixation (Yokota *et al.*, 1997; Bartová *et al.*, 2000; Volpi *et al.*, 2000; Galiová *et al.*, 2004). For example, Galiová *et al.* (2004) found that the β-like gene cluster was located at a radial position 56%-58% from the nuclear radius for 2D fixation and 62%-64% for 3D fixed cells. Kosubek *et al.* (2000) showed that preferential location of *ABL* and *BCR* genes was detected in both dehydrated and hydrated preparations. Thus a high number of nuclei analyzed in 2D may provide information regarding 3D structure, as was the case in the current study through the analysis of about 200 CTs (~100 nuclei).

According to other studies, the peripheral positioning of some genes in their CTs or their looping from out of these have been observed, although there are also reports showing internal positioning within CTs. For example, in four different human cell types, Scheuermann *et al.* (2005) showed that both genes and non-transcribed sequences were predominantly within the periphery of the respective chromosome territories, independent of transcriptional status and GC contents.

The location of genes in a large chromatin loop extending outside the chromosome territory has been displayed in the major human histo-compatibility complex (MHC) region of chromosome 6 territory (Volpi *et al.*, 2000) and in the ß-like globin gene cluster of chromosome 11 territory (Galiová *et al.*, 2004), and which were increased in frequency by cell stimulation.

Volpi *et al.* (2000) related their findings to the number of active genes in the MHC region, while Galiová *et al.* (2004) suggested that this phenomenon probably reflects the level of expression of the ß-like globin gene cluster.

In human breast cancer cell lines, thin chromatin protrusions carrying amplified *HER-2/neu* sequences and which extend from the painted chromosome 17 territory have also been found (Park and DeBoni, 1998). In human lymphomas, Roix *et al.* (2003) observed that the *CCND1* gene is frequently located in the interior of the nucleus, whereas others (c*-MYC, BCL6* and *IGK*) are preferentially located near the periphery. These authors suggested that in human gene loci, distribution patterns are frequently non-random and gene-specific in the interphase nucleus.

These observations seem to indicate that active genes might be found not only on the surface of the territory but also within. Chromosome territories are permeable to proteins and contain a large accessible internal surface created by a meshwork of interconnected channels (Parada *et al.*, 2002), thus there are no significant zones of exclusion within the nucleus itself.

Although preferential positioning of *CCND1* and *HER-2/neu* sequences at the periphery of the CTs has been observed in two types of tumor, our data did not reveal a relationship between gene positioning and the amplification and over-expression status of these genes. To the best of our knowledge, this is the first study that involved an evaluation of the association between the location of *CCND1* and *HER-2/neu* oncogenes and their amplification and over-expression status in esophageal and gastric cancers. In their previous report on breast tumor cell lines, Park and De Boni (1998) did not even relate the positioning of the *HER-2/neu* gene with alterations in the copy number of chromosome 17. As in our sample, tumors with amplification of the *HER-2/neu* gene presented non-uniformity in signal size, thereby suggesting possible clustering. However, we did not observe elongated protrusions of the signal extending outside chromosome 17 territory, although these authors did.

The *CCND1* and *HER-2/neu* genes, mapped to chromosome bands 11q13 (Motokura and Arnold, 1993) and 17q11.2-q12 (Yamamoto *et al.*, 1986), respectively, are both located in an R-band (early-replication region), believed to contain more transcriptional active genes than G-bands (Kurz *et al.*, 1996; Volpi *et al.*, 2000). Thus, our findings reinforce the hypothesis that R-bands are predominantly located in the periphery of the CTs (Volpi *et al.*, 2000).

The results strongly support the hypothesis that genes are preferentially positioned in the periphery of the CTs for transcription to occur. However other features such as gene density, DNA transcriptional level in G-band and R-band (gene-rich) regions, chromatin structure, gene characteristics, chromosomal structure, expression level and stability and tissue expression specificity, should also be considered (Volpi *et al.*, 2000; Kosak and Groudine, 2002).

In conclusion, despite inherent restrictions in the analysis procedure and limitations in methodology, our results suggest preferential positioning of *CCND1* and *HER-2/neu* oncogenes in the periphery of chromosome territories, in tumoral tissues of both the esophagus and stomach, probably without any association with amplification or over-expression of these genes. These findings emphasize the idea that factors other than gene transcriptional activity must be acting together. Therefore, further studies are needed to elucidate the relevance of gene positioning in interphase chromosome territories and differential gene expression.

## Figures and Tables

**Table 1:** Results on the location of *CCND1* and *HER-2/neu* genes in chromosome territories, and amplification and protein over-expression in normal and tumor tissues of the esophagus.

	*CCND1*						*HER-2/neu*				
Samples	Position in CT			Amplification (G/C>2.0)	Over- expression (> 30%)		Position in CT			Amplification (G/C>2.0)	Over- expression (2+, 3+)
	I (%)	P (%)	Category				I (%)	P (%)	Category		
Normal mucus											
MNE 01	65.1	34.9	I	0.9	35.4		63	37	I	1.0	0
MNE 02	24	75.8	P	1.0	25.2		68.5	31.4	I	0.9	0
MNE 03	76.5	23.4	I	0.9	25.4		74.2	25.8	I	0.9	0
MNE 04	36.2	63.7	P	0.9	20.4		67.3	32.6	I	0.9	0
MNE 05	19.7	80.3	P	0.9	12.2		59.7	40.2	I	1.0	0
( $\bar{X}$ ± s)	44.30 ± 25.26	55.62 ± 25.24					66.54 ± 5.53	33.4 ± 5.51			

Esophageal carcinoma											
CE 01	3	97	P	**9.6**	20.4		22	77.9	P	1.0	1+
CE 02	2.9	96.9	P	**9.3**	22.2		20	79.9	P	1.0	0
CE 03	67.1	32.8	I	0.9	10.6		56	44	I	0.7	0
CE 04	77.2	22.7	I	0.9	11.8		11.2	88.7	P	**8.9**	0
CE 05	2.9	96.9	P	**9.4**	**36.6**		71.7	28.3	I	1.0	1+
CE 06	74	25.4	I	1.0	2.0		45.5	54.5	P	0.9	0
CE 07	74.6	25.4	I	**3.5**	22.3		12.2	87.7	P	**2.3**	3+
CE 08	25	75	P	0.9	32.0		71.7	28.2	I	**2.4**	0
CE 09	27.9	72.1	P	1.8	0		46.1	54	P	0.9	0
CE 10	38.8	61.3	P	**4.2**	**49.0**		35.4	64.6	P	0.9	3+
( $\bar{X}$ ± s)	39.34 ± 31.55	60.55 ± 31.36					39.18 ± 22.78	60.78 ± 22.75			
p value	0.5033						0.0100				

Abbreviations: CT: chromosome territories; I: internal; P: peripheral; G/C: gene-to-centromere ratio.

**Table 2:** Results on location of *CCND1* and *HER-2/neu* genes in chromosome territories, and amplification and protein over-expression in normal and tumor tissues of the stomach.

	*CCND1*						*HER-2/neu*				
Samples	Position in CT			Amplification (G/C>2.0)	Over- expression (> 20%)		Position in CT			Amplification (G/C>2.0)	Over- expression (2+, 3+)
	I (%)	P (%)	Category				I (%)	P (%)	Category		
Normal mucus											
MNG 01	46	54	P	1.0	14.2		24.6	75.4	P	0.9	0
MNG 02	25	75	P	1.0	8.8		31	69	P	0.9	0
MNG 03	76.3	23.7	I	0.9	7.6		33	67	P	1.0	0
MNG 04	76.7	23.2	I	0.9	6.1		42	58	P	0.9	0
MNG 05	24.6	75.3	P	0.9	12.8		73.6	26.4	I	0.9	0
( $\bar{X}$ ± s)	49.72 ± 25.93	50.24 ± 25.93					40.84 ± 19.34	59.16 ± 19.34			

Gastric cancer											
CG 01	12.2	87.7	P	1.5	22.5		36.6	63.4	P	0.9	0
CG 02	7.4	92.4	P	1.0	5.6		60	40	I	1.7	2+
CG 03	47.3	52.6	P	0.9	5.2		34.8	65.1	P	0.9	0
CG 04	1.6	98.3	P	0.9	25.6		24.6	75.2	P	0.9	0
CG 05	9	90.9	P	1.0	20.2		29.8	70.1	P	1.0	0
CG 06	15	85	P	1.0	27.6		19.3	80.5	P	1.0	0
CG 07	25.9	74	P	1.0	17.2		38.7	61.3	P	0.9	0
CG 08	17.9	82.1	P	0.9	35.0		25	74.9	P	1.0	0
CG 09	12.3	87.6	P	1.0	0.0		25.4	74.5	P	0.9	0
CG 10	13.2	86.7	P	0.9	12.0		37.8	62.2	P	0.9	0
( $\bar{X}$ ± s)	16.18 ± 12.68	83.73 ± 12.67					33.20 ± 11.50	66.72 ± 11.44			
p value	< 0.0001						0.0017				

Abbreviations: CT: chromosome territories; I: internal; P: peripheral; G/C: gene-to-centromere ratio.

**Table 3:** Relationship between gene location (peripheral or internal) of *CCND1* in chromosome 11 territory, and amplification and over-expression in esophageal carcinoma and gastric adenocarcinoma.

Samples/ positions	Amplification (FISH)				Over-expression (IHC)		
	*CCND1* (+)	*CCND1* (-)	Total		Cyclin D1 (+)	Cyclin D1 (-)	Total
Esophageal carcinoma							
Peripheral	4 (40%)	2 (20%)	6 (60%)		3 (30%)	3 (30%)	6 (60%)
Internal	1 (10%)	3 (30%)	4 (40%)		0 (0%)	4 (40%)	4 (40%)
Total	5 (50%)	5 (50%)	10 (100%)		3 (30%)	7 (70%)	10 (100%)
Kappa	0.400*				0.444*		
p value	0.088				0.091		

Gastric cancer							
Peripheral	0 (0%)	10 (100%)	10 (100%)		5 (50%)	5 (50%)	10 (100%)
Internal	0 (0%)	0 (0%)	0 (0%)		0 (0%)	0 (0%)	0 (0%)
Total	0 (0%)	10 (100%)	10 (100%)		5 (50%)	5 (50%)	10 (100%)
Kappa	a				a		
p value	a				a		

*Value of Kappa = 0.40-0.59: moderated agreement. a value of Kappa or p not estimated. FISH: Fluorescence *in situ* hybridization. IHC: immunohistochemical.

**Table 4:** Relationship between gene location (peripheral or internal) of *HER-2/ neu* in chromosome 17 territory, and amplification and over-expression, in esophageal carcinoma and gastric adenocarcinoma.

Samples/ positions	Amplification (FISH)				Over-expression (IHC)		
	*HER-2/neu* (+)	*HER-2/neu* (-)	Total		Her-2/neu (+)	Her-2/neu (-)	Total
Esophageal Carcinoma							
Peripheral	2 (20%)	5 (50%)	7 (70%)		2 (20%)	5 (50%)	7 (70%)
Internal	1 (10%)	2 (20%)	3 (30%)		0 (0%)	3 (30%)	3 (30%)
Total	3 (30%)	7 (70%)	10 (100%)		2 (20%)	8 (80%)	10 (100%)
Kappa	-0.034*				0.194**		
p value	a				0.301		

Gastric cancer							
Peripheral	0 (0%)	9 (90%)	9(90%)		0 (0%)	9 (90%)	9 (90%)
Internal	0 (0%)	1 (10%)	1 (10%)		1 (10%)	0 (0%)	1 (10%)
Total	0 (0%)	10 (100%)	10 (100%)		1 (10%)	9 (90%)	10 (100%)
Kappa	a				-0.22*		
p value	a				a		

*Value of Kappa < 0: no agreement. **Value of Kappa = 0-0.19: slightpoor agreement. a value of Kappa or p not estimated. FISH: Fluorescence *in situ* hybridization. IHC: immunohistochemical.
